# How immune breakthroughs could slow disease progression and improve prognosis in COVID-19 patients: a retrospective study

**DOI:** 10.3389/fimmu.2023.1246751

**Published:** 2023-10-23

**Authors:** Yiting Wang, Bennan Zhao, Xinyi Zhang, Xia Zhang, Fengjiao Gao, Xiaoyan Yuan, Xiaoxia Ren, Maoquan Li, Dafeng Liu

**Affiliations:** ^1^ The First Ward of Internal Medicine, Public Health Clinic Centre of Chengdu, Chengdu, China; ^2^ School of Public Health, Chengdu Medical College, Chengdu, China; ^3^ Department of Endocrinology & Metabolism, Sichuan University West China Hospital, Chengdu, China

**Keywords:** coronavirus disease 2019 (COVID-19), immune breakthroughs, vaccination, previous infection, disease progression, prognosis

## Abstract

**Background:**

Previous infections and vaccinations have produced preexisting immunity, which differs from primary infection in the organism immune response and may lead to different disease severities and prognoses when reinfected.

**Objectives:**

The purpose of this retrospective cohort study was to investigate the impact of immune breakthroughs on disease progression and prognosis in patients with COVID-19.

**Methods:**

A retrospective cohort study was conducted on 1513 COVID-19 patients in Chengdu Public Health Clinical Medical Center from January 2020 to November 2022. All patients were divided into the no immunity group (primary infection and unvaccinated, n=1102) and the immune breakthrough group (previous infection or vaccination, n=411). The immune breakthrough group was further divided into the natural immunity subgroup (n=73), the acquired immunity subgroup (n=322) and the mixed immunity subgroup (n=16). The differences in clinical and outcome data and T lymphocyte subsets and antibody levels between two groups or between three subgroups were compared by ANOVA, t test and chi-square test, and the relationship between T lymphocyte subsets and antibody levels and the disease progression and prognosis of COVID-19 patients was assessed by univariate analysis and logistic regression analysis.

**Results:**

The total critical rate and the total mortality rate were 2.11% and 0.53%, respectively. The immune breakthrough rate was 27.16%. In the no immunity group, the critical rate and the mortality rate were all higher, and the coronavirus negative conversion time was longer than those in the immune breakthrough group. The differences in the critical rate and the coronavirus negative conversion time between the two groups were all statistically significant (3.72% vs. 0.24%, 14.17 vs. 11.90 days, all p<0.001). In addition, in the no immunity group, although lymphocyte counts and T subsets at admission were higher, all of them decreased consistently and significantly and were significantly lower than those in the immune breakthrough group at the same time from the first week to the fourth week after admission (all p<0.01). The total antibody levels and specific Immunoglobulin G (IgG) levels increased gradually and were always significantly lower than those in the immune breakthrough group at the same time from admission to the fourth week after admission (all p<0.001). Moreover, in the natural immunity subgroup, lymphocyte counts and T subsets at admission were the highest, and total antibody levels and specific IgG levels at admission were the lowest. Then, all of them decreased significantly and were the lowest among the three subgroups at the same time from admission to one month after admission (total antibody: from 546.07 to 158.89, IgG: from 6.00 to 3.95) (all p<0.001). Those in the mixed immunity subgroup were followed by those in the acquired immunity subgroup. While lymphocyte counts and T subsets in these two subgroups and total antibody levels (from 830.84 to 1008.21) and specific IgG levels (from 6.23 to 7.51) in the acquired immunity subgroup increased gradually, total antibody levels (from 1100.82 to 908.58) and specific IgG levels (from 7.14 to 6.58) in the mixed immunity subgroup decreased gradually. Furthermore, T lymphocyte subsets and antibody levels were negatively related to disease severity, prognosis and coronavirus negative conversion time. The total antibody, specific IgM and IgG levels showed good utility for predicting critical COVID-19 patients and dead COVID-19 patients.

**Conclusion:**

Among patients with COVID-19 patients, immune breakthroughs resulting from previous infection or vaccination, could decelerate disease progression and enhance prognosis by expediting host cellular and humoral immunity to accelerate virus clearance, especially in individuals who have been vaccinated and previously infected.

**Clinical trial registry:**

Chinese Clinical Trial Register ChiCTR2000034563.

## 1 Introduction

Since mid-December 2019, the COVID-19 pandemic caused by SARS-CoV-2 has been ongoing and evolved into a major global health threat (1). By April 2023, over 762 million confirmed cases and over 6.8 million deaths have been reported globally (2). SARS-CoV-2 elicits both innate and adaptive immune responses, including the development of specific T cells and antibodies. Efficient immune responses are indispensable for the regulation and eradication of pathogen infections (3).

The adaptive immune system consists of three lymphocyte types: B cells, CD3+CD4+ T cells, and CD3+CD8+ T cells (4). T-lymphocytes and immune antibodies are necessary to control viral infections. Similar to severe influenza and other respiratory viral infections, lymphopenia is frequently observed in COVID-19 and exhibits a positive correlation with the clinical disease severity (5, 6). Adaptive responses of immune antibodies provide the first line of defense during viral infections and are important for long-term immunity and immune memory (7). Therefore, the number of T cells and antibodies can be diagnostic and predictive factors for identifying patients who will have severe disease (6, 8).

The topic of pre-existing immunity to SARS-CoV-2 infection, acquired through natural infection or vaccination, has gained significant attention currently (9). Several countries have also reported cases of breakthrough infections among individuals who were vaccinated or had a previous infection (9–11). Studies have shown that the maintenance of IgG and T-cell responses persists in most patients for at least 3–4 months following infection (12) and even more than 13 months (13). Vaccination is considered key to reducing the risk of SARS-CoV-2 infection, severe illness, and mortality risks (14). The immune breakthrough is speculated to be attributed to factors including high viral load exposure, infection with a different viral strain, and antibody-dependent enhancement (10). In the post-COVID-19 era, understanding the potential influence of immune breakthrough is crucial to improving COVID-19 prevention and control measures. Therefore, we conducted a population-based study to investigate the presence of SARS-CoV-2 T lymphocytes and antibody levels, carefully examining the fluctuations in these levels among patients with varying immune statuses.

## 2 Methods

### 2.1 Subjects

**Figure 1:**
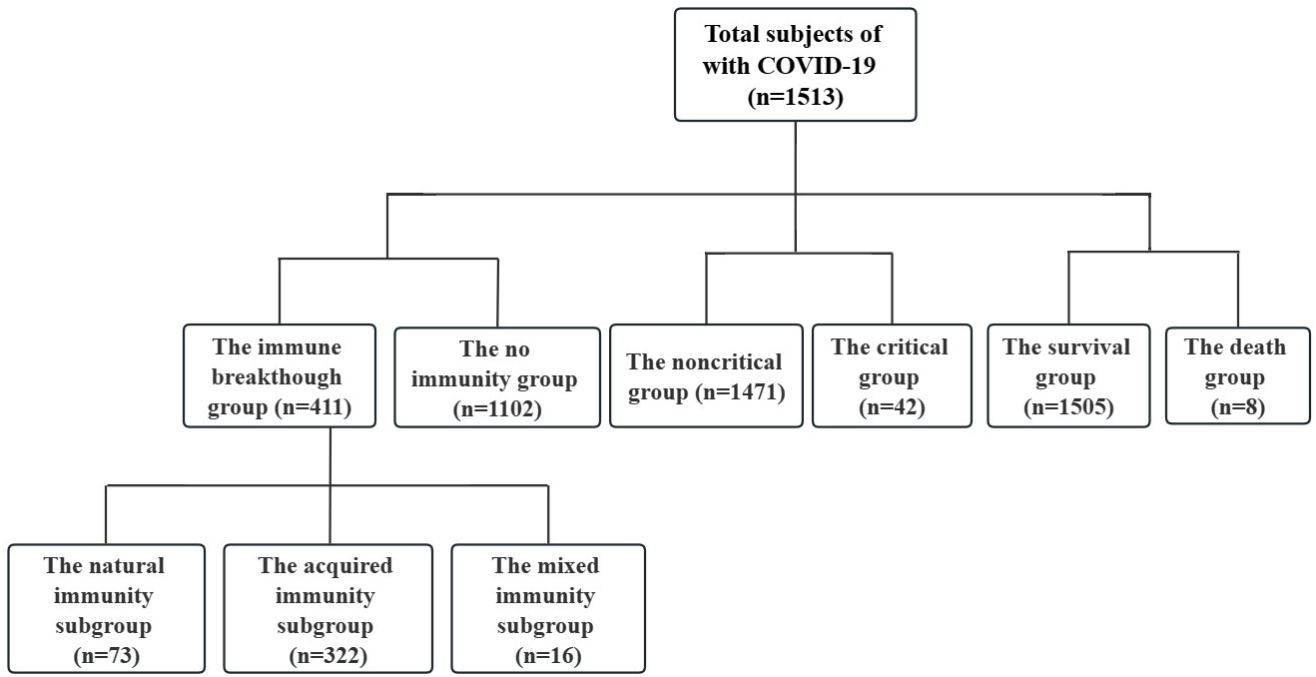
Patient data (*n*=1513). Non critical refers to the clinical type of COVID-19 that is asymptomatic, light and common. Critical refers to the clinical type of COVID-19 that is associated with severe and critical illness. No immunity refers to primary infection and no vaccination. Immune breakthrough refers to previous infection or vaccination. Natural immunity refers to previous infection. Acquired immunity refers to vaccination. Mixed immunity refers to previous infection and vaccination.

This was a retrospective cohort study. All 1,513 patients with COVID-19 from the hospital isolation ward who presented to the Public Health Clinical Centre of Chengdu from January 16, 2020, to September 30, 2022, were retrospectively recruited (**Figure 1**, **Table 1**). The study was approved by the Public and Health Clinical Centre of Chengdu Ethics Committee (ethics approval number: PJ-K2020-26-01). Written informed consent was waived by the Ethics Commission of the designated hospital because this study was related to emerging infectious diseases.

**Table 1:** Baseline information (*n*=1513).

Variables	
Age(year), [M (IQR)]	35.0(27.0-47.0)
Male, n (%)	1074(71.0)
Female, n (%)	438(29.0)
BMI, [M (IQR)]	23.34(20.81-26.03)
Duration of hospitalization (day), [M (IQR)]	15.0(11.0-20.0)
The coronavirus negative conversion time (day), [M (IQR)]	11.0(6.0-18.0)
Disease severity	
Noncritical illness, n (%)	1471(97.2)
Critical illness, n (%)	42(2.8)
Number of comorbidities	
0, n (%)	547(36.6)
1, n (%)	374(25.0)
2, n (%)	233(15.6)
3 or more, n (%)	340(22.8)
Source of cases	
Domestically transmitted cases, n (%)	227(15.0)
Imported cases, n (%)	1283(85.0)
Prognosis	
Survive, n (%)	1505(99.5)
Death, n (%)	8(0.5)
Infection and vaccination status	
Immune breakthrough, n (%)	411(27.2)
No immunity, n (%)	1102(72.8)
Dose of vaccination	
1 dose, n (%)	13(0.9)
2 doses, n (%)	235(15.5)
3 doses, n (%)	84(5.6)
4 doses, n (%)	6(0.4)

BMI, body mass index.

### 2.2 Inclusion and exclusion criteria

The inclusion criteria were as follows: no sex limit; age ≥18 years old; COVID-19; and inpatient isolation and treatment time >1 day.

The exclusion criteria were as follows: age<18 years old and isolation and treatment time <1 day.

### 2.3 Disease diagnosis, clinical typing, cure criteria and laboratory testing

The criteria for COVID-19 clinical typing, disease diagnosis and cure were in accordance with the seventh Trial Version of the Novel Coronavirus Pneumonia Diagnosis and Treatment Guidance (15).

The diagnosis criteria were cases with one of the following etiological pieces of evidence: real-time fluorescence reverse transcription-polymerase chain reaction (RT-PCR) detected the positive nucleic acid of the new coronavirus and sequencing of viral genes.

The typing criteria were as follows: (1) asymptomatic infection indicated that there were no clinical symptoms and no pneumonia manifestations on imaging; (2) the light type indicated that the clinical symptoms were mild, and there were no pneumonia manifestations on imaging; (3) the common type indicated that the clinical symptoms included fever and respiratory tract, and pneumonia could be seen on imaging; (4) the severe type indicated that the patients had any of the following criteria: respiratory distress, RR≥30 times/min; in the resting state, oxygen saturation ≤ 93%; arterial blood oxygen partial pressure (PaO2)/oxygen concentration (FiO2)≤300mmHg (1mmHg=0.133kPa), living in areas with high altitude (over 1000 meters above sea level), and PaO2/FiO2 should be corrected according to the following formula: PaO2/FiO2*[atmospheric pressure(mmHg)/760]; pulmonary imaging showed that lesions with significant progress over 50% within 24–48h were managed as heavy; (5) the critical illness type criteria included one of the following conditions: respiratory failure occurs and mechanical ventilation is needed; Shock occurs; and combining other organ failure requires intensive care units (ICU) monitoring.

The cured discharge standard was as follows: the body temperature returned to normal for more than 3 days; respiratory symptoms improved significantly; lung imaging showed a significant improvement in acute exudative lesions; and two consecutive sputum, nasopharyngeal swabs and other respiratory specimens tested negative for nucleic acid (sampling time at least 24 h apart).

The relevant serological assays were performed by Enzyme-Linked Immunosorbent Assay (ELISA) in the laboratory of the hospital.

### 2.4 Grouping standards

Among the 1,513 COVID-19 cases, 1102 and 411 cases were divided into the no immunity group (primary infection and no vaccination) and the immune breakthrough group (previous infection or vaccination), respectively (**Figure 1**, **Table 1**).

Among the 411 immune breakthrough cases, 73 patients with previous infection were assigned to the natural immunity group, 322 patients who had been vaccinated (no distinction was made between vaccine type and dose) were assigned to the acquired immunity group, and 16 patients who had both previous infection and been vaccinated were assigned to the mixed immunity group (**Figure 1**, **Table 1**).

Among the 1,513 COVID-19 cases, 1,471 noncritical patients (patients with asymptomatic infection, with light and with common clinical type) were assigned to the noncritical group, and 42 critical patients (patients with severe and with critical illness clinical type) were assigned to the critical group (**Figure 1**, **Table 1**).

Among the 1,513 COVID-19 cases, 1505 surviving patients were assigned to the survival group, and 8 dead patients were assigned to the death group (**Figure 1**, **Table 1**).

### 2.5 Definition of the viral negative conversion time, disease severity and prognosis

The disease severity included critical illness (COVID-19 patients with severe or critical illness clinical type) and noncritical illness (COVID-19 patients with asymptomatic infection, light or common clinical type). The prognosis included death and survival within four weeks after admission. The coronavirus negative conversion time was the time from onset to the first negative nucleic acid test meeting the discharge criteria.

### 2.6 Data collection

The data were collected from a subset of patients treated at Chengdu Public Health Clinical Medical Center from January 2020 to November 2022. All data of 1,513 cases, including clinical data, laboratory data and demographic data, were collected to establish databases. Researchers strictly controlled the accuracy, completeness and authenticity of all data.

### 2.7 Statistical analysis

SPSS 26.0 (SPSS, Chicago, IL, USA) and GraphPad Prism 8 (GraphPad, CA, USA) were used for statistical analyses. Measurement data with a normal distribution are presented as the mean and standard deviation, and measurement data with a nonnormal distribution are presented as the median and interquartile range (IQR). The categorical data are expressed as a percentage or proportion. Data with a normal distribution and homogeneity of variance between multiple groups were compared using one-way or two-way ANOVA, and further comparison between two groups was performed using the least significant difference (LSD) t test. Data with a normal distribution and homogeneity of variance between two groups were compared using the independent samples t test. Enumeration data are presented as percentages or proportions, and data between two or multiple groups were compared using a chi-square test. Analysis of influencing factors of disease severity and prognosis was performed using binary logistic regression analysis. Receiver operating characteristic (ROC) analysis was used to assess lymphocytes and subsets to distinguish non critical from severe COVID-19 patients. P<0.05 was considered statistically significant.

### 2.8 Patient and public involvement

Patients and the public were not involved in the development of the research questions or in the design of the study. Patients received verbal and written information about the study; however, they were not involved in the recruitment of subjects or the conduct of the study. Additionally, the burden of the intervention was assessed by the investigators. The participants were assessed for eligibility, and data collection was performed. Dissemination of the general results (without personally identifying data) will occur on demand. The Ethics Committee of the Public Health Clinical Centre of Chengdu approved this study (ethics approval number: PJ-K2020-26-01). Written informed consent was waived by the Ethics Commission of the designated hospital because this study is related to emerging infectious diseases.

## 3 Results

### 3.1 Baseline conditions (characteristics of the study population)

A total of 1,513 patients with COVID-19 were included in this study. Their demographic and clinical characteristics are listed in **Table 1**. The median age of all patients included in the study was 35 years, and males accounted for the majority (71.0%). The median coronavirus negative conversion time was 11.0 days, and the duration of hospitalization was 15.0 days.

In addition, 966 (63.40%) patients had comorbidities, 374 (25.0%) patients had one comorbidity, 233 (15.6%) patients had two comorbidities, 340 (22.8%) patients had three or more comorbidities, and 547 (36.6%) patients had no comorbidities. Among them, 42 (2.8%) patients had critical illness, 1,471 (97.2%) patients had noncritical illness, 1505 patients survived, and only 8 (0.5%) patients died.

Imported cases accounted for 85.0% of the total, while domestically transmitted cases made up the remaining 15.0%. Regarding immune status, 411 (27.2%) patients had preexisting immunity by previous infection or vaccination, and 1102 (72.8%) patients had primary infection. Among vaccinated patients, 13 (0.9%) patients accepted one dose, 235 (15.5%) patients accepted two doses, 84 (5.6%) patients accepted three doses and 6 (0.4%) patients accepted four doses.

### 3.2 Comparisons between the immune breakthrough group and the no immunity group

In the immune breakthrough group, the proportion of domestically transmitted cases was significantly higher than that in the no immunity group (**Table 2**) (p<0.05), while the critical illness rate was significantly lower than that in the no immunity group (**Table 2**) (0.24% vs. 3.72%, p<0.05). The mortality rate was slightly lower than that in the no immunity group, although the difference was not statistically significant (**Table 2**) (p=0.083).

**Table 2:** Comparison of baseline conditions between the two groups (n=1513).

Variables	immune breakthrough (n=411)	no immunity (n=1102)	χ2	*P*
Gender			10.603	0.005
Male, n (%)	269(65.5)	805(73.0)		
Female, n (%)	142(34.5)	297(27.0)		
Number of comorbidities			4.073	0.539
0, n (%)	153(38.2)	394(36.1)		
1, n (%)	99(24.7)	275(25.2)		
2, n (%)	60(15.0)	173(15.8)		
3 or more, n (%)	89(22.2)	251(23.0)		
Disease severity			13.411	0.000
Noncritical illness, n (%)	410(99.8)	1061(96.3)		
Critical illness, n (%)	1(0.2)	41(3.7)		
Source of cases			17.051	0.000
Imported cases, n (%)	373(90.8)	910(82.6)		
Domestically transmitted cases, n (%)	36(9.3)	191(17.4)		
Prognosis			3.000	0.083
Survive, n (%)	411(100)	1094(99.3)		
Death, n (%)	0(0)	8(0.7)		

**Figure 2:**
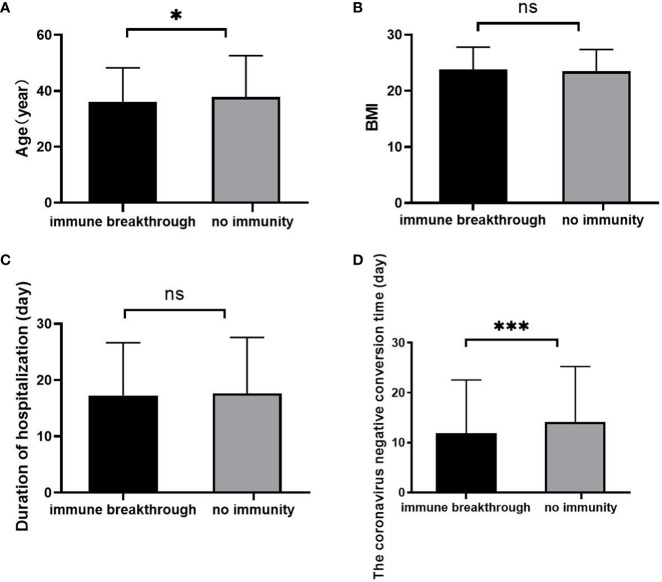
Comparison of age, BMI, duration of hospitalization and coronavirus negative conversion time between the immune breakthrough group and the no immunity group (n=1513; the immune breakthrough group and the no immunity group, n=411 and 1102, respectively). **(A)** Age. **(B)** BMI. **(C)** Duration of hospitalization (day). **(D)** The coronavirus negative conversion time (day). BMI, body mass index. Unpaired *t* tests were used for comparisons between two groups, ^ns^p>0.05, *p<0.05, ***p<0.001.

The age (**Figure 2A**) was slightly younger than that in the no immunity group (p<0.05), and there was no significant difference in BMI (**Figure 2B**) or duration of hospitalization (**Figure 2C**) between the two groups (all p>0.05). However, in the immune breakthrough group, the coronavirus negative conversion time (**Figure 2D**) was significantly shorter than that in the no immunity group (p<0.001).

**Figure 3:**
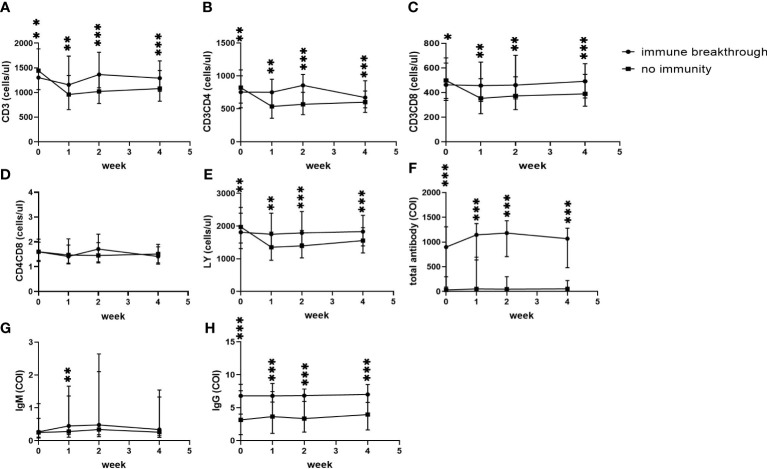
Comparison of lymphocyte counts, T subset counts and antibody levels between the immune breakthrough group and the no immunity group within 4 weeks (*n*=1513; the immune breakthrough group and the no immunity group, *n*=411 and 1102, respectively). LY, lymphocyte; IgM, immunoglobulin M; IgG, immunoglobulin G; **(A)** CD3+ counts. **(B)** CD3+CD4+ counts. **(C)** CD3+CD8+ counts. **(D)** CD4+CD8+ counts. **(E)** LY. **(F)** Total antibody levels. **(G)** IgM levels. **(H)** IgG levels. Two-way ANOVA was used for intergroup comparisons within 4 weeks (**A–H**, *P* all<0.01). Unpaired *t* tests were used for comparisons between two groups at the same time point, *p<0.05, **p<0.01, ***p<0.001.

In addition, in the no immunity group, CD3+ counts, CD3+CD4+ counts, CD3+CD8+ counts and lymphocyte counts at admission were higher than those in the immune breakthrough group (**Figures 3A–C, E**) (all p<0.05), but all of them then decreased to the lowest level at the first week and were always significantly lower than those in the immune breakthrough group from the first week to the fourth week (**Figures 3A–C, E**) (all p<0.01). The ratio of CD3+CD4+ to CD3+CD8+ cells between the two groups from onset to the fourth week after onset was always similar to each other (**Figure 3D**) (p>0.01). Moreover, in the no immunity group, total antibody levels and specific IgG levels from onset to the fourth week were always significantly lower than those in the immune breakthrough group (**Figures 3F, H**) (all p<0.001). In the no immunity group, the specific IgM levels were always lower than those in the immune breakthrough group from the first week to the fourth week, but a significant difference was found only at the first week after onset (**Figure 3G**).

### 3.3 Comparison of types of comorbidities between the immune breakthrough group and the no immunity group

Metabolic diseases such as fatty liver, diabetes, hypertension, hyperlipidemia, etc. were the most common in both groups, with no difference (p all <0.05) (**Table 3**). The incidence of cardiovascular disease was higher in the no immunity group (p<0.05) (**Table 3**). Besides, the incidence of hypoxemia (p<0.001) and cardiovascular disease (p<0.05) was significantly higher in the no immunity group than in the immune breakthrough group (**Table 3**).

**Table 3:** Comparison of types of comorbidities between the two groups (n=1513).

Comorbidities	immune breakthrough (n=411)	no immunity (n=1102)	χ2	*P*
Diabetes n (%)	19(20.9)	72(79.1)	1.933	0.164
Hypertension n (%)	34(22.5)	117(77.5)	1.832	0.176
Hypoxemia n (%)	0(0)	43(100)	16.506	0.000
Hyperlipidemia n (%)	54(24.1)	170(75.9)	1.242	0.265
Hyperuricemia n (%)	31(32.0)	66(68.0)	1.204	0.273
Hypokalemia n (%)	26(29.2)	63(70.8)	0.201	0.654
Leukopenia n (%)	2(10.0)	18(90.0)	3.018	0.082
Chronic obstructive pulmonary disease n (%)	1(4.8)	20(95.2)	5.402	0.020
Hepatitis B n (%)	14(19.7)	57(80.3)	2.088	0.148
Metabolic associated fatty liver disease n (%)	89(24.0)	281(76.0)	2.395	0.122
Kidney stones n (%)	10(24.4)	31(75.6)	0.164	0.686
Cardiovascular Diseases n (%)	2(8.3)	22(91.7)	4.371	0.037
Anemia n (%)	5(29.4)	12(70.6)	0.044	0.834

### 3.4 Comparisons between the natural immunity subgroup, the acquired immunity subgroup and the mixed immunity subgroup

Compared to the natural immunity subgroup and the acquired immunity subgroup, age was slightly younger (p<0.05), and the duration of hospitalization was obviously shorter than that in the mixed immunity subgroup (p<0.01) (**Table 4**). The coronavirus negative conversion time in the mixed immunity subgroup was also slightly shorter than that in the other two groups, but the difference was not statistically significant (**Table 4**) (p=0.057).

**Table 4:** Comparison of baseline conditions between the three groups.

Variables	natural immunity(n=73)	acquired immunity(n=322)	mixed immunity(n=16)	F/χ2	*P*
Male, n (%)	51(69.9)	206(64.8)	12(75.0)	1.274	0.529
Age(year)	36.79	36.30	28.75	3.128	0.045
BMI	23.97	23.76	23.15	0.285	0.752
The coronavirus negative conversion time (day)	10.85	12.44	6.44	2.884	0.057
Duration of hospitalization (day)	14.11	18.19	13.00	7.610	0.001
Disease severity				0.227	0.871
Noncritical illness, n (%)	73(100)	321(99.7)	16(100)		
Critical illness, n (%)	0(0)	1(0.3)	0(0)		

**Figure 4:**
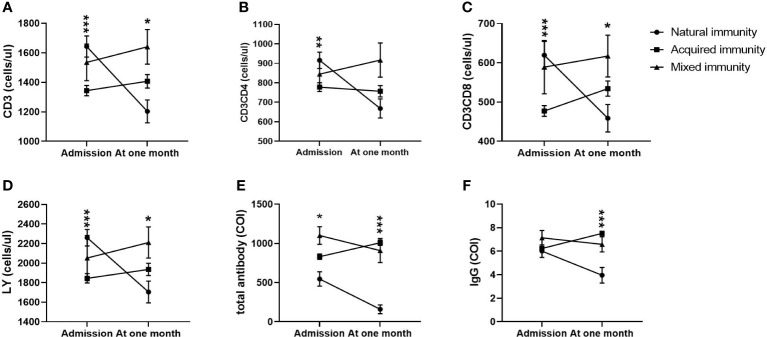
Comparison of lymphocyte counts, T subset counts and antibody levels among the natural immunity subgroup, the acquired immunity subgroup and the mixed immunity subgroup within 4 weeks (*n*=411; the natural immunity subgroup, the acquired immunity subgroup and the mixed immunity subgroup, *n*=73, 322 and 16, respectively). LY, lymphocyte; IgM, immunoglobulin M; IgG, immunoglobulin G. **(A)** CD3+ counts. **(B)** CD3+CD4+ counts. **(C)** CD3+CD8+ counts. **(D)** LY. **(E)** Total antibody levels. **(F)** IgG levels. Two-way ANOVA was used for intergroup comparisons within 4 weeks (**A–F**, *P* all<0.01). Unpaired *t* tests were used for comparisons between two groups at the same time point, *p<0.05, **p<0.01, ***p<0.001.

Moreover, in the acquired immunity subgroup, CD3+ counts, CD3+CD4+ counts, CD3+CD8+ counts and lymphocyte counts at admission were the highest and then decreased to the lowest level at the first month among the three groups. All of them in the other two groups showed an increase from admission to the first month after admission, and those in the mixed immunity group reached their highest levels (**Figures 4A–D**). In the mixed immunity subgroup, the total antibody levels and specific IgG levels were the highest at admission and then decreased slightly over the following month (**Figures 4E, F**). Those in the natural immunity subgroup were the lowest at admission, with a sharp decrease during the following month (**Figures 4E, F**). Only those in the mixed immunity subgroup rose from second place on admission to first place at one month (**Figures 4E, F**).

### 3.5 Univariate and multivariate analysis of baseline characteristics, lymphocyte subsets, and antibody levels for disease severity and prognosis

For critical cases, the age was significantly older, the number of comorbidities and the imported cases were higher (p all=0.000). Most T-lymphocyte subsets (except CD3+CD8+% and CD19+%) and all antibody levels (p all<0.05) were significantly higher in critical cases, whereas the level of CD56+% (p=0.024) was higher in non-critical cases (**Table 5**). Similarly, all the death cases were imported cases, with an average age of 77.5 years (p=0.000), a slightly higher proportion of females (p=0.034), an increased BMI index (p=0.013), and all combined with more than 3 diseases (p=0.000), without a history of previous infection. CD3+, CD3+CD4+, CD3+CD8+, LY, LY%, CD19+ and all antibody levels in non-critical cases were higher (p all <0.01) (**Table 5**).

**Table 5:** Univariate analysis of disease severity and prognosis.

Variables	Disease severity				Prognosis			
	non critical (n=1471)	critical (n=42)	t/χ2	p	cured (n=1505)	death (n=8)	t/χ2	*P*
Age (year)	36.73	57.86	-10.263	0.000	37.11	77.50	-8.578	0.000
Gender			0.456	0.499			4.495	0.034
Male	1046(97.4)	28(2.6)			1071(99.7)	3(0.3)		
Female	425(96.8)	14(3.2)			439(98.9)	5(1.1)		
BMI	23.52	24.84	-2.060	0.134	23.54	27.49	-2.479	0.013
Number of comorbidities			96.530	0.000			27.614	0.000
0, n (%)	546(99.6)	2(0.4)			547(100.0)	0(0)		
1, n (%)	382(99.5)	2(0.5)			384(100.0)	0(0)		
2, n (%)	239(98.4)	4(1.6)			243(100.0)	0(0)		
3 or more	304(89.9)	34(10.1)			331(97.6)	8(2.4)		
Source of cases			180.545	0.000			45.457	0.000
Imported cases, n (%)	190(83.7)	37(16.3)			219(96.5)	8(3.5)		
Domestically transmitted cases, n (%)	1278(99.6)	5(0.4)			1286(100.0)	0(0)		
Previous infection	89(100.0)	0(0)	2.700	0.100	89(100.0)	0(0)	0.503	0.478
CD3+	1445.30	593.40	8.627	0.000	1426.08	443.75	4.333	0.000
CD3+CD4+	829.29	326.95	10.216	0.000	817.86	256.63	4.050	0.000
CD3+CD8+	517.48	232.27	6.869	0.000	511.13	167.38	3.615	0.000
CD3+%	72.41	62.27	3.505	0.004	72.30	70.46	0.685	0.493
CD3+CD4+%	41.55	37.09	3.734	0.002	41.43	40.75	0.251	0.802
CD3+CD8+%	28.97	26.79	0.161	0.872	28.92	26.21	0.089	0.929
LY	1990.77	872.36	8.147	0.000	1965.72	643.38	4.185	0.000
LY%	22.58	9.87	1.671	0.000	22.29	7.89	0.836	0.000
CD19+	214.33	83.64	3.658	0.000	204.35	73.83	1.730	0.006
CD56+	187.93	116.14	2.520	0.012	182.12	126.00	0.941	0.348
CD19+%	12.40	11.21	1.230	0.220	12.35	8.93	1.708	0.089
CD56+%	12.10	15.29	-2.356	0.024	12.33	16.27	-1.396	0.164
Total antibody	504.47	80.88	1.717	0.005	502.26	30.23	0.832	0.003
IgM	1.64	0.05	0.604	0.005	1.64	0.01	0.308	0.006
IgG	5.30	2.00	1.735	0.042	5.29	1.01	1.393	0.007

LY, lymphocyte.

The influencing factors for disease severity by multiple linear regression analysis were age, lymphocyte percentage, source of cases and CD3+CD8+ counts (**Table 6**). Moreover, the influencing factors for prognosis were age, sex, and source of cases (**Table 6**).

**Table 6:** Multiple stepwise regression analysis of influencing factors of disease severity and prognosis (n=95).

independent variable		B	Std. Error	Beta	t	*P*
the disease severity	constant	0.753	0.083	–	9.041	0.000
	age	-0.005	0.001	-0.264	-4.608	0.000
	LY (%)	0.014	0.003	0.3968	4.852	0.000
	Source of cases	0.124	0.035	0.205	3.590	0.000
	CD3+CD8+(cells/ul)	0.000	0.000	-0.236	-2.892	0.004
the prognosis	constant	1.007	0.015	–	65.130	0.000
	age	-0.001	0.000	-0.150	-5.575	0.000
	Source of cases	0.018	0.006	0.086	3.161	0.002
	gender	-0.010	0.004	-0.059	-2.207	0.027

BMI, body mass index; LY, lymphocyte.

### 3.6 The prediction of the antibody levels on disease severity and prognosis in patients with COVID-19

**Figure 5:**
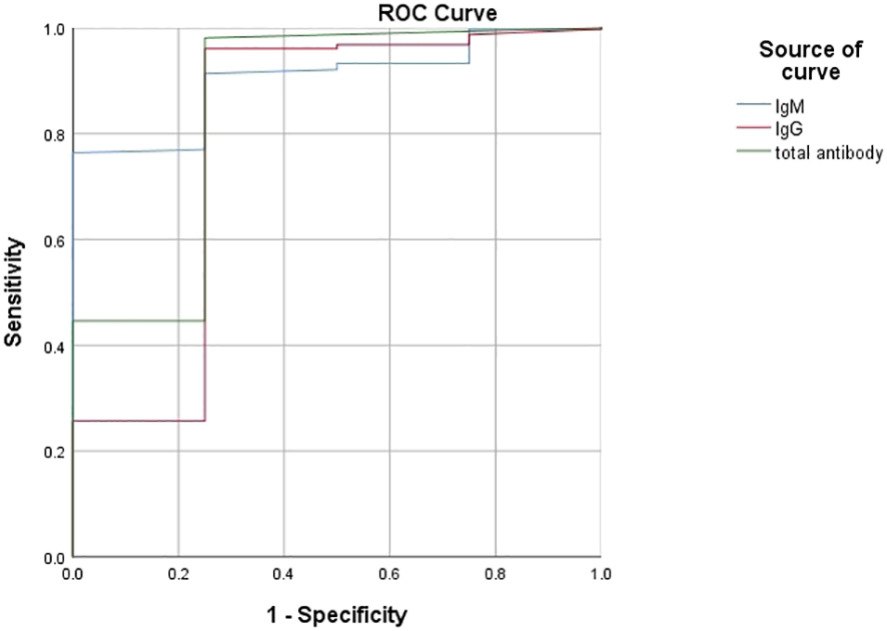
Using characteristics of antibody levels for discriminating the critical cases from the noncritical patients (*n*=1513; critical and noncritical groups, *n*=42 and 1471, respectively). ROC analysis showing the performance of antibody levels in distinguishing critical cases from noncritical patients. ROC, receiver operating characteristic curve.

According to the ROC analysis, the total antibody, IgM and IgG levels showed good utility for predicting critical COVID-19 patients (**Table 7**). The areas under the curve of total antibody, IgM and IgG for disease severity were 0.854, 0.904 and 0.794, respectively (**Table 7**, **Figure 5**). The sensitivities were 98.10%, 76.40%, 96.10%, while the specificities were 75.00%, 100.00%, 75.00% (**Table 7**).

**Table 7:** The performance of various methods for distinguishing between critical cases and non-critical cases (n=1513).

variables	Cutoff point	AUC (95%CI)	Sensitivity	Specificity	False positive	False negative
Total antibody	0.015	0.854(0.623~1.000)	98.10%	75.00%	1.90%	25.00%
IgM	0.0905	0.904(0.819~0.988)	76.40%	100.00%	23.60%	0.00%
IgG	0.0085	0.794(0.619~1.000)	96.10%	75.00%	3.90%	25.00%

AUC, area under the curve; CI, confidence interval.

## 4 Discussion

In the post epidemic era, breakthrough infections of SARS-CoV-2 are being increasingly observed worldwide due to the high pervasiveness of viral spread, the emergence of novel variants (11), progressive ease of restrictive measures and waning protection against infection. In this retrospective study, we found that the critical illness rate was 2.8%, and the mortality rate was 0.5%, both of which were low, which was consistent with our previous report (16–19). We also found that the immune breakthrough rate in this study cohort was 27.2%, with 4.8% of patients having previous infections, 22.4% of patients having been vaccinated, and only 6% of patients having been fully vaccinated. This finding align with previous reports indicating that 4.0% to 5.3% of fully vaccinated individuals exhibit immune dysfunction (20, 21). A previous study reported that among fully vaccinated individuals, the incidence rate for COVID-19 breakthrough infection was 5.0 per 1000 person-months (20). Compared with partial vaccination, the incidence rate for COVID-19 breakthrough infection in full vaccination was associated with a 28% reduced risk (20). The reinfection rate among patients with COVID-19 is estimated to range from 2.3% to 21.4%, as indicated by a meta-analysis (22). The reinfection rate in our study was also in the range.

We reported the differences in disease severity and prognosis and in T lymphocyte subsets and antibody levels between different immune statuses to investigate the impact of immune breakthroughs on disease progression and prognosis in patients with COVID-19. We found that patients with preexisting immunity by previous infection or vaccination had a lower severe rate and mortality rate than those with primary infection, with all fatalities occurring exclusively among patients without previous immune protection. The results align with the findings reported in the existing literature (23–25). Since the hypoxemia secondary to infection occurred in the no immunity group, the proportion of patients with chronic obstructive pulmonary disease and cardiovascular disease was also higher in the no immunity group, suggesting that the infection combined with cardiopulmonary system diseases may be the main factor for the development of severe illness. Many studies have reported that patients with preexisting immunity have milder illnesses and lower rates of hospitalization (23, 24). Immune breakthrough patients were significantly less likely to experience severe disease or death than matched unvaccinated patients (25). With only one severe case and no deaths in preexisting immunity patients, we were unable to analyze differences in severe and mortality rates according to route of immunization. However, it also implies that pre-existing immunity diminishes the probability of progressing to severe illness following reinfection.

Similar to previous studies (3, 6), we observed higher levels of T-lymphocytes and antibodies in non-critical cases. We also found that patients with preexisting immunity by previous infection or vaccination exhibited shorter coronavirus negative conversion times and higher T lymphocyte subsets and antibody levels. SARS-CoV-2 infection is associated with lymphopenia, particularly in CD4+ T cells and CD8+ T cells (26), indicating abnormal immune function during SARS-CoV-2 infection (27). Failure to generate a timely T-cell response during natural SARS-CoV-2 infection has been linked to the development of severe COVID-19 cases (4). The immune memory generated by vaccination or natural infection serves as a reservoir of protective immunity that can rapidly expand upon re-exposure to the virus, potentially limiting viral replication during the early stages of infection (28). In addition to mitigating the severity of infection, breakthrough infections are less infectious than primary infections (29).

Preexisting immunity is associated with four major components of immunological memory: memory CD4+ T cells, memory CD8+ T cells, antibodies, and memory B cells (4). Both SARS-CoV-2-specific CD4+ and CD8+ memory T cells peaked within the initial month of infection, followed by a gradual decline over the subsequent 6 to 7 months (30). The CD4+ and CD8+ T-cell responses exhibited comparable levels between mRNA vaccination and infection, with similar T-cell production observed at both 6 months after the second dose and 6 months post-infection (31). Patricia (32) et al. found that the peak T-cell response 2 weeks after full vaccination was comparable to the peak response in mild and moderate patients. However, varying vaccine types, doses and intervals resulted in different immune memory statuses (31). In a retrospective study from Israel, in individuals who received the Pfizer mRNA vaccine (never infected), higher initial antibody levels were followed by a more rapid decline than in those with SARS-CoV-2 virus infection (33). Failure to generate sufficient IgG antibodies is linked to reduced survival (34). In contrast to our findings, some studies showed higher levels of antibodies (IgM and IgG) in severe patients than in mild-to-moderate patients (35–37). These results indicated that, in addition to antiviral efficacy, antibody responses might be associated with secondary antibody-mediated organ damage (36). Due to our subjects were mainly non critical patients, the lymphocyte and antibody levels of critical patients were not analyzed.

In addition, we further analyzed the different immune statuses of the three preexisting immune routes: natural immunity, acquired immunity and mixed immunity. The T-lymphocyte subsets exhibited the highest level one month after infection, surpassing that of the other two groups. Total antibody and IgG levels were the highest at admission and decreased slowly over the following month. Levels of anti-S1 IgG production were much higher in vaccinated individuals than in naturally infected individuals within three months of vaccination. Many studies have shown that vaccination induces a more robust and targeted immune response compared to the natural infection (32, 38). Therefore, the optimal approach to combat COVID-19 is to enhance immune function through vaccination. However, many studies have shown that mixed immunization appears to confer a more robust protective effect (39, 40). Our study also confirmed this view, with the shortest length of hospitalization and the shortest coronavirus negative conversion time results in the mixed immunization group (**Table 4**). A single dose of vaccine elicited higher memory T and B-cell responses in previously infected individuals (41). Due to the existence of immune memory, previously infected individuals produce more rapid and lasting cellular and humoral immunity after vaccination (42, 43). Further research found that there were significantly higher levels of antibodies in fully vaccinated individuals with natural immunity than in fully vaccinated individuals without prior infection. Vaccination after previous infection appeared to enhance and prolong immunity, even more than 1 year after the initial infection, with no sign of weakening (39). These studies suggest that in the post pandemic era, we can still fight the virus through a combination of natural immunity and vaccination.

The association between advanced age, a high number of comorbidities, and an unfavorable prognosis has been observed in previous study (16). The higher mortality rate observed in domestically transmitted cases is expected to be attributed to the limited extent of domestic transmission during the period of case collection. The large difference in the number of critical and noncritical patients (1471 vs 42) may have biased the results of the ROC analysis, but in combination with the results of the univariate analysis, it can be inferred that the antibody level is a reliable indicator of prognosis. Consistent with previous findings (5, 6, 8), critical and dead cases exhibited lower levels of T-lymphocyte subsets and antibodies, which served as reliable indicators for disease progression.

However, there are still some limitations of this study. Importantly, it was a single-center, retrospective study, and all the inherent limitations of retrospective studies are unavoidable. The incidence of critical cases, especially deaths, was minimal. Additionally, reinfected cases were extensively interviewed after a time interval, allowing the possibility of recall bias. Due to the information restrictions, the data related to the strains are in the CDC, which we cannot get. We can only infer from the time that the strains of our patients’ infection range from the original strain SARS-CoV-2 to Delta variant (B.1.617.2) and omicron (B.1.1.529). In addition, vaccine types were not available and the sequencing of vaccination and infection was not distinguished, no further analysis could be conducted.

## 5 Conclusion

Currently, reported cases do not accurately represent the infection rate due to a global reduction in testing and reporting, but the World Health Organization still reports more than 1 million new positive cases every four weeks. Hence, our findings serve as a valuable reference for predicting disease progression and treating patients with COVID-19 by monitoring changes in lymphocyte subsets and antibodies. The immune breakthrough group had lower rates of critical disease and mortality compared to the no immunity group, while the mixed immunity group showed highest levels of T-lymphocyte subsets and antibodies. Therefore, vaccination should be intensified to enhance the protective effect even in the post pandemic era, when most people have already been infected.

## Data availability statement

The raw data supporting the conclusions of this article will be made available by the authors, without undue reservation.

## Ethics statement

The study was approved by the Public and Health Clinical Centre of Chengdu Ethics Committee (ethics approval number: PJ-K2020-26-31 01). Written informed consent was waived by the Ethics Commission of the designated hospital because this study was related to emerging infectious diseases.

## Author contributions

Concept and design: YW, BZ, XinZ, XiaZ, FG, XY, XR, ML, DL; Data acquisition: YW, BZ, XinZ, XiaZ, FG, XY, XR, ML, DL; data analysis and interpretation: YW, BZ, XinZ, XiaZ, FG, XY, XR, ML, DL; Drafting manuscript: YW, BZ, XinZ, XiaZ, FG, XY, XR, ML, DL; administrative, technical, or material support: YW, BZ, XinZ, XiaZ, FG, XY, XR, ML, DL; study supervision: DL. All authors contributed to the article and approved the submitted version.

## References

[1] Du Toit A. Outbreak of a novel coronavirus. Nat Rev Microbiol (2020) 18(3):123–. doi: 10.1038/s41579-020-0332-0 PMC7073251 31988490

[2] World Health O. Covid-19 Weekly Epidemiological Update, Edition 138, 13 April 2023. Geneva: World Health Organization (2023).

[3] Zhang P Du W Yang T Zhao L Xiong R Li Y. Lymphocyte subsets as a predictor of severity and prognosis in Covid-19 patients. Int J Immunopathology Pharmacol (2021) 35:20587384211048567. doi: 10.1177/20587384211048567 PMC8504275 34619994

[4] Sette A Crotty S. Adaptive immunity to Sars-Cov-2 and Covid-19. Cell (2021) 184(4):861–80. doi: 10.1016/j.cell.2021.01.007 PMC7803150 33497610

[5] Zhao Q Meng M Kumar R Wu Y Huang J Deng Y. Lymphopenia is associated with severe Coronavirus Disease 2019 (Covid-19) infections: A systemic review and meta-analysis. Int J Infect Dis (2020) 96:131–5. doi: 10.1016/j.ijid.2020.04.086 PMC7196544 32376308

[6] Pan P Du X Zhou Q Cui Y Deng X Liu C. Characteristics of lymphocyte subsets and cytokine profiles of patients with Covid-19. Virol J (2022) 19(1):57. doi: 10.1186/s12985-022-01786-2 35346253 PMC8960102

[7] Racine R Winslow GM. Igm in microbial infections: taken for granted? Immunol Lett (2009) 125(2):79–85. doi: 10.1016/j.imlet.2009.06.003 19539648 PMC2747358

[8] Vengesai A Midzi H Kasambala M Mutandadzi H Mduluza-Jokonya TL Rusakaniko S. A systematic and meta-analysis review on the diagnostic accuracy of antibodies in the serological diagnosis of Covid-19. Systematic Rev (2021) 10(1):155. doi: 10.1186/s13643-021-01689-3 PMC8152206 34039423

[9] Nordström P Ballin M Nordström A. Risk of Sars-Cov-2 reinfection and Covid-19 hospitalisation in individuals with natural and hybrid immunity: A retrospective, total population cohort study in Sweden. Lancet Infect Dis (2022) 22(6):781–90. doi: 10.1016/S1473-3099(22)00143-8 PMC8971363 35366962

[10] Tillett RL Sevinsky JR Hartley PD Kerwin H Crawford N Gorzalski A. Genomic evidence for reinfection with Sars-Cov-2: A case study. Lancet Infect Dis (2021) 21(1):52–8. doi: 10.1016/S1473-3099(20)30764-7 PMC7550103 33058797

[11] Wang J Kaperak C Sato T Sakuraba A. Covid-19 reinfection: A rapid systematic review of case reports and case series. J Invest Med (2021) 69(6):1253–5. doi: 10.1136/jim-2021-001853 34006572

[12] Jiang XL Wang GL Zhao XN Yan FH Yao L Kou ZQ. Lasting antibody and T cell responses to Sars-Cov-2 in Covid-19 patients three months after infection. Nat Commun (2021) 12(1):897. doi: 10.1038/s41467-021-21155-x 33563974 PMC7873066

[13] Kucinskaite-Kodze I Simanavicius M Simaitis A Zvirbliene A. Persistence of Sars-Cov-2-specific antibodies for 13 months after infection. Viruses-Basel (2021) 13(11):2123. doi: 10.3390/v13112313 PMC8622371 34835119

[14] Kent SJ Khoury DS Reynaldi A Juno JA Wheatley AK Stadler E. Disentangling the relative importance of T cell responses in Covid-19: leading actors or supporting cast? Nat Rev Immunol (2022) 22(6):387–97. doi: 10.1038/s41577-022-00716-1 PMC9047577 35484322

[15] Diagnosis and treatment protocol for novel coronavirus pneumonia (Trial Version 7). Chin Med J (Engl) (2020) 133(9):1087–95. doi: 10.1097/CM9.0000000000000819 PMC7213636 32358325

[16] Liu DF Zheng YL Kang J Wang DM Bai L Mao Y. Not only high number and specific comorbidities but also age are closely related to progression and poor prognosis in patients with Covid-19. Front Med (2022) 8:736109. doi: 10.3389/fmed.2021.736109 PMC8782432 35071254

[17] Liu DF Wang Y Zhao BN Lan LJ Liu YL Bao L. Overall reduced lymphocyte especially T and B subsets closely related to the poor prognosis and the disease severity in severe patients with Covid-19 and diabetes mellitus. Diabetol Metab Syndrome (2021) 13(1):5. doi: 10.1186/s13098-020-00622-3 PMC7802992 33436069

[18] Zhou YY Chen Y Zhang XY Zhao BN Gao FJ Yuan XY. Nutritional risk and a high Nrs2002 score are closely related to disease progression and poor prognosis in patients with Covid-19. Front Nutr (2023) 10:1089972. doi: 10.3389/fnut.2023.1089972 37125047 PMC10130536

[19] Liu DF Lan LJ Luo DX Zhao BN Wei G He YS. Lymphocyte subsets with the lowest decline at baseline and the slow lowest rise during recovery in Covid-19 critical illness patients with diabetes mellitus. Diabetes Res Clin Pract (2020) 167:108341. doi: 10.1016/j.diabres.2020.108341 32707212 PMC7373679

[20] Sun J Zheng QL Madhira V Olex AL Anzalone AJ Vinson A. Association between immune dysfunction and Covid-19 breakthrough infection after Sars-Cov-2 vaccination in the us. JAMA Internal Med (2022) 182(2):153–62. doi: 10.1001/jamainternmed.2021.7024 PMC8715386 34962505

[21] Khoury J Najjar-Debbiny R Hanna A Jabbour A Abu Ahmad Y Saffuri A. Covid-19 vaccine-long term immune decline and breakthrough infections. Vaccine (2021) 39(48):6984–9. doi: 10.1016/j.vaccine.2021.10.038 PMC8556595 34763949

[22] Piri SM Edalatfar M Shool S Jalalian MN. Tavakolpour S. A systematic review on the recurrence of Sars-Cov-2 virus: frequency, risk factors, and possible explanations. Infect Dis (2021) 53(5):315–24. doi: 10.1080/23744235.2020.1871066 PMC7852280 33508989

[23] Pilz S Theiler-Schwetz V Trummer C Krause R Ioannidis JPA. Sars-Cov-2 reinfections: overview of efficacy and duration of natural and hybrid immunity. Environ Res (2022) 209:112911. doi: 10.1016/j.envres.2022.112911 35149106 PMC8824301

[24] Fabianova K Kyncl J Vlckova I Jirincova H Kostalova J Liptakova M. Covid-19 reinfections. Epidemiologie Mikrobiologie Imunologie (2021) 70(1):62–7. 33853339

[25] Butt AA Nafady-Hego H Chemaitelly H Abou-Samra AB Al Khal A Coyle PV. Outcomes among Patients with Breakthrough Sars-Cov-2 Infection after Vaccination. Int J Infect Dis (2021) 110:353–8. doi: 10.1016/j.ijid.2021.08.008 PMC8349447 34375762

[26] Vafaeinezhad A Atashzar MR Baharlou R. The immune responses against coronavirus infections: friend or foe? Int Arch Allergy Immunol (2021) 182(9):863–76. doi: 10.1159/000516038 PMC8247827 33951640

[27] Bobcakova A Petriskova J Vysehradsky R Kocan I Kapustova L Barnova M. Immune profile in patients with Covid-19: lymphocytes exhaustion markers in relationship to clinical outcome. Front Cell Infection Microbiol (2021) 11:646688. doi: 10.3389/fcimb.2021.646688 PMC8082075 33937096

[28] Jordan SC. Innate and adaptive immune responses to Sars-Cov-2 in humans: relevance to acquired immunity and vaccine responses. Clin Exp Immunol (2021) 204(3):310–20. doi: 10.1111/cei.13582 PMC8013613 33534923

[29] Abu-Raddad LJ Chemaitelly H Ayoub HH Tang P Coyle P Hasan MR. Relative infectiousness of Sars-Cov-2 vaccine breakthrough infections, reinfections, and primary infections. Nat Commun (2022) 13(1):532. doi: 10.1038/s41467-022-28199-7 35087035 PMC8795418

[30] Cohen KW Linderman SL Moodie Z Czartoski J Lai LL Mantus G. Longitudinal analysis shows durable and broad immune memory after Sars-Cov-2 Infection with persisting antibody responses and memory B and T cells. Cell Rep Med (2021) 2(7):100354. doi: 10.1016/j.xcrm.2021.100354 34250512 PMC8253687

[31] S ette A Crotty S. Immunological memory to Sars-Cov-2 infection and Covid-19 vaccines. Immunol Rev (2022) 310(1):27–46. doi: 10.1111/imr.13089 35733376 PMC9349657

[32] Almendro-Vazquez P Laguna-Goya R Ruiz-Ruigomez M Utrero-Rico A Lalueza A de la Calle GM. Longitudinal dynamics of Sars-Cov-2-specific cellular and humoral immunity after natural infection or Bnt162b2 vaccination. PloS Pathog (2021) 17(12):e1010211. doi: 10.1371/journal.ppat.1010211 34962970 PMC8757952

[33] Israel A Shenhar Y Green I Merzon E Golan-Cohen A Schaeffer AA. Large-scale study of antibody titer decay following Bnt162b2 mrna vaccine or Sars-Cov-2 infection. Vaccines (2022) 10(1):64. doi: 10.3390/vaccines10010064 PMC8781423 35062724

[34] Nairz M Sahanic S Pizzini A Bohm A Tymoszuk P Mitterstiller AM. Quantity of igg response to Sars-Cov-2 spike glycoprotein predicts pulmonary recovery from Covid-19. Sci Rep (2022) 12(1):3677. doi: 10.1038/s41598-022-07489-6 35256646 PMC8901626

[35] Liu XT Zheng X Liu B Wu MX Zhang ZL Zhang GC. Serum Igm against Sars-Cov-2 Correlates with in-Hospital Mortality in Severe/Critical Patients with Covid-19 in Wuhan, China. Aging-Us (2020) 12(13):12432–40. doi: 10.18632/aging.103417 PMC7377873 32628642

[36] Zhang BC Zhou XY Zhu CL Song YX Feng F Qiu YR. Immune phenotyping based on the neutrophil-to-lymphocyte ratio and igg level predicts disease severity and outcome for patients with Covid-19. Front Mol Biosci (2020) 7:157. doi: 10.3389/fmolb.2020.00157 32719810 PMC7350507

[37] Korobova ZR Zueva EV Arsentieva NA Batsunov OK Liubimova NE Khamitova IV. Changes in anti-Sars-Cov-2 igg subclasses over time and in association with disease severity. Viruses-Basel (2022) 14(5):941. doi: 10.3390/v14050941 PMC9143443 35632683

[38] Samanovic MI Oom AL Cornelius AR Gray-Gaillard SL Karmacharya T Tuen M. Vaccine-acquired Sars-Cov-2 immunity versus infection-acquired immunity: A comparison of three Covid-19 vaccines. Vaccines (2022) 10(12):2152. doi: 10.3390/vaccines10122152 36560562 PMC9782527

[39] Hall V Foulkes S Insalata F Kirwan P Saei A Atti A. Protection against Sars-Cov-2 after Covid-19 Vaccination and Previous Infection. New Engl J Med (2022) 386(13):1207–20. doi: 10.1056/NEJMoa2118691 PMC8908850 35172051

[40] Vicenti I Basso M Gatti F Scaggiante R Boccuto A Zago D. Faster decay of neutralizing antibodies in never infected than previously infected healthcare workers three months after the second Bnt162b2 mrna Covid-19 vaccine dose. Int J Infect Dis (2021) 112:40–4. doi: 10.1016/j.ijid.2021.08.052 PMC8410637 34481967

[41] Sasikala M Shashidhar J Deepika G Ravikanth V Krishna VV Sadhana Y. Immunological memory and neutralizing activity to a single dose of Covid-19 vaccine in previously infected individuals. Int J Infect Dis (2021) 108:183–6. doi: 10.1016/j.ijid.2021.05.034 PMC8132551 34022331

[42] Gobbi F Buonfrate D Moro L Rodari P Piubelli C Caldrer S. Antibody response to the Bnt162b2 mrna Covid-19 vaccine in subjects with prior Sars-Cov-2 infection. Viruses-Basel (2021) 13(3):422. doi: 10.3390/v13030422 PMC8001674 33807957

[43] Bagno FF Andrade LAF Sergio SAR Parise PL Toledo-Teixeira DA Gazzinelli RT. Previous infection with Sars-Cov-2 correlates with increased protective humoral responses after a single dose of an inactivated Covid-19 vaccine. Viruses-Basel (2022) 14(3):510. doi: 10.3390/v14030510 PMC8955604 35336917

